# The New Hospital for Women

**Published:** 1902-07-12

**Authors:** 


### THE YEAR'S WORK.

### THE NEW HOSPITAL FOR WOMEN.

### THE YEAR'S WORK.

The work was somewhat interrupted during 1901 owing to the necessity for repainting some of the wards and renewing the kitchen range. For three weeks the hospital was emptied, and for two weeks the out-patient department was closed. Consequently only 573 in-patients were treated, 215 being medical, 337 surgical, and 21 ophthalmic. There were, however, 141 major operations, while in 1900 there were only 115. There were 23 deaths; six were due to malignant disease. In the out-patient department, notwithstanding the closing, there were nearly 1,000 more new patients than in 1900, the total number of visits being 34,863. The difficulty of organising this large number with justice both to physician and patient has several times occupied the committee during the year, and it has been decided to limit the number of new cases each day to 25. Except by doubling or trebling the size of this department, it is inevitable that there should be disappointments, and on one occasion nearly 60 had the fatigue and expense of the journey for nothing. Each of these would gladly have paid 6d. to consult a medical woman. In the eye department 651 new cases have been seen, and in the children's department 250. Two hundred and eight maternity patients were attended in their own homes. Over 1,900 have now been attended, and the only maternal death was due to apoplexy. Forty-two students have taken their training in vaccination. The total receipts were £5,882 15s. 4d., and the total expenditure £5,768 17s. 11½d.; £700 of the receipts, however, was invested by desire, and when all bills were paid there was a deficit of £387. Patients' payments amounted to £1,543 14s. 2d. A fund is being raised for a memorial in London to the late Emperor and Empress Frederick; this will take the form of the endowment of a bed for patients suffering from cancer, and the promoters have decided to place this memorial bed in the New Hospital; £2,000 is required, and contributions have already been received from Sir J. Lauder Brunton, Lady Priestly, the Dowager Lady Jenner and others. The hon. secretary of the fund is Miss L. A. Jenner, 39 Addison Road, W. A letter on the subject, signed by Lady Frances Balfour, the Bishop of Ripon and others, appeared in the *Times* of March 26th, 1902.

